# Development and validation of a digital biopsy model to predict microvascular invasion in hepatocellular carcinoma

**DOI:** 10.3389/fonc.2024.1360936

**Published:** 2024-09-17

**Authors:** Emrullah Birgin, Heiner Nebelung, Schaima Abdelhadi, Johann S. Rink, Matthias F. Froelich, Svetlana Hetjens, Mohammad Rahbari, Patrick Téoule, Erik Rasbach, Christoph Reissfelder, Jürgen Weitz, Stefan O. Schoenberg, Carina Riediger, Verena Plodeck, Nuh N. Rahbari

**Affiliations:** ^1^ Department of General and Visceral Surgery, University Hospital Ulm, Ulm, Germany; ^2^ Department of Radiology, Faculty of Medicine and University Hospital Carl Gustav Carus, Technische Universität Dresden, Dresden, Germany; ^3^ Department of Surgery, Universitätsmedizin Mannheim, Medical Faculty Mannheim, Heidelberg University, Mannheim, Germany; ^4^ Department of Radiology and Nuclear Medicine, University Medical Centre Mannheim, University of Heidelberg, Mannheim, Germany; ^5^ Department of Medical Statistics and Biomathematics, Medical Faculty Mannheim, Heidelberg University, Mannheim, Germany; ^6^ DKFZ Hector Cancer Institute at the University Medical Center Mannheim, Mannheim, Germany; ^7^ Department of Visceral-, Thoracic and Vascular Surgery, Faculty of Medicine and University Hospital Carl Gustav Carus, Technische Universität Dresden, Dresden, Germany

**Keywords:** biomarker, radiology, resection, perioperative oncology, hepatectomy

## Abstract

**Background:**

Microvascular invasion is a major histopathological risk factor of postoperative recurrence in patients with hepatocellular carcinoma. This study aimed to develop and validate a digital biopsy model using imaging features to predict microvascular invasion before hepatectomy.

**Methods:**

A total of 217 consecutive patients who underwent hepatectomy for resectable hepatocellular carcinoma were enrolled at two tertiary-care reference centers. An imaging-based digital biopsy model was developed and internally validated using logistic regression analysis with adjustments for age, sex, etiology of disease, size and number of lesions.

**Results:**

Three imaging features, i.e., non-smoothness of lesion margin (OR = 16.40), ill-defined pseudocapsula (OR = 4.93), and persistence of intratumoral internal artery (OR = 10.50), were independently associated with microvascular invasion and incorporated into a prediction model. A scoring system with 0 - 3 points was established for the prediction model. Internal validation confirmed an excellent calibration of the model. A cutoff of 2 points indicates a high risk of microvascular invasion (area under the curve 0.87). The overall survival and recurrence-free survival stratified by the risk model was significantly shorter in patients with high risk features of microvascular invasion compared to those patients with low risk of microvascular invasion (overall survival: median 35 vs. 75 months, P = 0.027; recurrence-free survival: median 17 vs. 38 months, P < 0.001)).

**Conclusion:**

A preoperative assessment of microvascular invasion by digital biopsy is reliable, easily applicable, and might facilitate personalized treatment strategies.

## Introduction

Hepatocellular carcinoma (HCC) is a major global health challenge with a rising incidence worldwide (1). HCC development is closely related to chronic liver disease with viral hepatitis, alcoholic and non-alcoholic steatohepatitis as leading etiologies (1). Due to the reliance on tumor burden and the functional hepatic reserve for determining patient treatment and outcomes, managing HCC proves exceptionally challenging (2). The complexity of this heterogenous disease and its treatment is reflected in markedly variable outcomes following potentially curative therapy such as surgical resection, liver transplantation, or local ablation (3). Patients undergoing these treatments typically exhibit a 5-year survival rate of approximately 62-70% (4, 5). Moreover, HCC recurrence in patients after potentially curative treatment remains a major burden with rates up to 70% within 5 years after treatment (6). Microvascular invasion (MVI), characterized by the microscopic presence of tumor cells in hepatic vessels (arteries, hepatic vein, and portal vein) lined with endothelial cells, stands as the most crucial determinant of disease recurrence and long-term survival (7). Unfortunately, MVI can only be histopathologically diagnosed based on the resected surgical specimen and therefore its use to guide personalized treatment strategies remains limited. Recently, the prediction of MVI before surgery has gained increasing attention, with several promising noninvasive methods utilizing imaging features or tumor markers embedded into risk models (8–12). However, current evidence is primarily characterized by complex risk models involving multimodal biomarkers, or restriction of imaging modalities to either magnetic resonance imaging (MRI) or computed tomography (CT) (13, 14). Furthermore, the vast majority of risk models were developed in selective subsets of HCC patients (i.e., predominantly viral hepatitis) with significant imbalances of the number of predictor variables and high MVI rates in the cohorts hampering its transferability to the clinical routine (12, 15, 16).

In this study, we aimed to develop a noninvasive digital biopsy risk model to predict MVI using preoperative imaging features and assess its prognostic outcome in patients undergoing hepatectomy for HCC.

## Methods

### Study population

This retrospective cohort study was approved by the institutional review board (2023–831) and conducted in line with the Declaration of Helsinki and the Transparent Reporting of a Multivariable Prediction Model for Individual Prognosis or Diagnosis (TRIPOD) guidelines (17). All consecutive patients who underwent hepatectomy for HCC between April 2008 and June 2023 at the Department of Surgery, University Hospital Mannheim and Department of Gastrointestinal, Thoracic and Vascular Surgery, University Hospital Carl Gustav Carus at the Technische Universität Dresden were identified from prospectively recorded databases and screened for eligibility. Informed consent was obtained from each patient to store data on prospective databases. The following inclusion criteria were used: a) adult patients (age 18 years or older) who underwent hepatectomy in curative-intent for resectable HCC; b) histopathologically documented status of MVI; c) preoperative imaging including computed tomography (CT) and/or magnetic resonance imaging (MRI) within 3 months of surgery. We excluded patients who had an inadequate quality of imaging for the evaluation of imaging features associated with MVI, and patients with mixed-type HCC-cholangiocarcinoma.

### Definitions and data acquisition

Patient records were reviewed for clinical variables such as age, gender, underlying liver disease, presence of liver cirrhosis, Child-Pugh classification, preoperative treatment, preoperative laboratory values such as alanine-aminotransferase, aspartate-aminotransferase, albumin, bilirubin, platelet count, and international normalized ratio. Pathological data included the number of resected lesions (classified as single, oligonodular (2-3 lesions) or multinodular (> 3 lesions)) (18), the diameter of lesions, resection margin, and the presence of microvascular invasion. Operative details, including the type and extent of hepatectomy, were also extracted. The Brisbane classification was used to categorize liver resections (19). Major hepatectomy was defined as resections of four or more Couinaud segments. HCC lesions were considered for resection irrespective of lesion size if patients had resectable lesions (single or multifocal) with an adequate future liver remnant, liver function, and performance status as well as the absence of distant metastasis or portal vein thrombosis. Postoperative surveillance included routine abdominal multiphasic computed tomography or magnetic resonance imaging and chest radiography every three months. Dates of last follow-up, recurrence, and death were recorded to calculate overall- and recurrence-free survival from the time of hepatectomy. Recurrence-free survival was defined as the time from hepatectomy to the first documented disease recurrence (radiologic or histologic evidence of local, regional, or distant metastasis) or death by any cause.

### Imaging analysis

Preoperative CT and MRI images were independently evaluated by two radiologists at each center who were blinded to clinical, surgical, pathologic, and follow-up results. At each center, discordance between two radiologists was solved by a third senior radiologist until consensus was generated. The presence of the following radiologic markers was assessed for its potential association with MVI as previously described in the literature (8, 10, 16, 20, 21): 1. extrahepatic growth pattern, i.e., exophytic lesions; 2. intratumoral hemorrhage; 3. intratumoral necrosis; 4. intratumoral vascularity, i.e. hyper-arterial enhancement in the arterial phase within the tumor; 5. internal artery, i.e. persistence of intratumoral arterial enhancements in the portal phase; 6. ill-defined incomplete pseudocapsula, i.e., irregular peritumoral hyperenhancement on portal phase of a radiological tumor capsule; 7. nonsmooth margin, i.e., nodular lesions with extranodular growth, confluent multinodular lesions or focal infiltrative margins; 8. peritumoral halo, i.e., peritumoral hypodense or hypointense halo in the portal phase; 9. rim enhancement, i.e., irregular circumferential peritumoral enhancement in the arterial phase; 10. wedge-shaped lesion, i.e. peritumoral hypodense or hypointense lesion located outside of the tumor margin in the delayed or hepatobiliary phase.

### Reference standard

Microvascular invasion was defined as nests of tumor cells lining vascular cavities of endothelial cells or portal and hepatic systems on hematoxylin and eosin staining (22). To determine the histopathological MVI status, all specimens were analyzed by two independent pathologists blinded to the clinical outcomes at each center.

### Statistics

Data between the study groups were evaluated using the Fisher’s exact test for categorical data and t- or Mann-Whitney-U tests for continuous data. In the training cohort, a logistic regression analysis with adjustments for age, sex, underlying liver disease, size, and number of lesions was conducted to identify significant predictors of preoperative imaging features to predict MVI. Significant variables (P < 0.05) on univariate analysis were applied to a multivariate analysis while controlling for age, sex, underlying liver disease, size, and number of lesions to develop the digital biopsy prediction model. A scoring system was derived from the β regression coefficient values divided by 2 to the nearest integer and an optimal threshold for patients at high risk of MVI was determined by the Youden’s index. Two risk categories were defined (low versus high risk) and internally validated by 1,000 bootstrap samples (23). The model performance, predictive strength, and model accuracy were evaluated by the area under the curve (AUC), the Nagelkerke’s R^2^ (a value of 1 indicates perfect fit), and the Brier scores (a value of 0 indicates total accuracy) (23). The calibration performance of the model was visualized by a calibration plot. The Kaplan-Meier method was used to calculate survival outcomes (log-rank test). To estimate the sample size for adequate modeling (24), the presence of MVI in the training cohort was anticipated to be 15% (25) and the number of candidate predictors to be included in the multivariate model was restricted to three variables. Assuming an estimated input C-statistic of 0.95, a shrinkage factor of 0.9, and an optimism of 0.05 in the apparent R^2^, a minimum sample size of 196 patients were calculated. A two-sided p-value < 0.05 was considered statistically significant. Statistical analyses were performed using R version 4.1.2.

## Results

**Figure 1:**
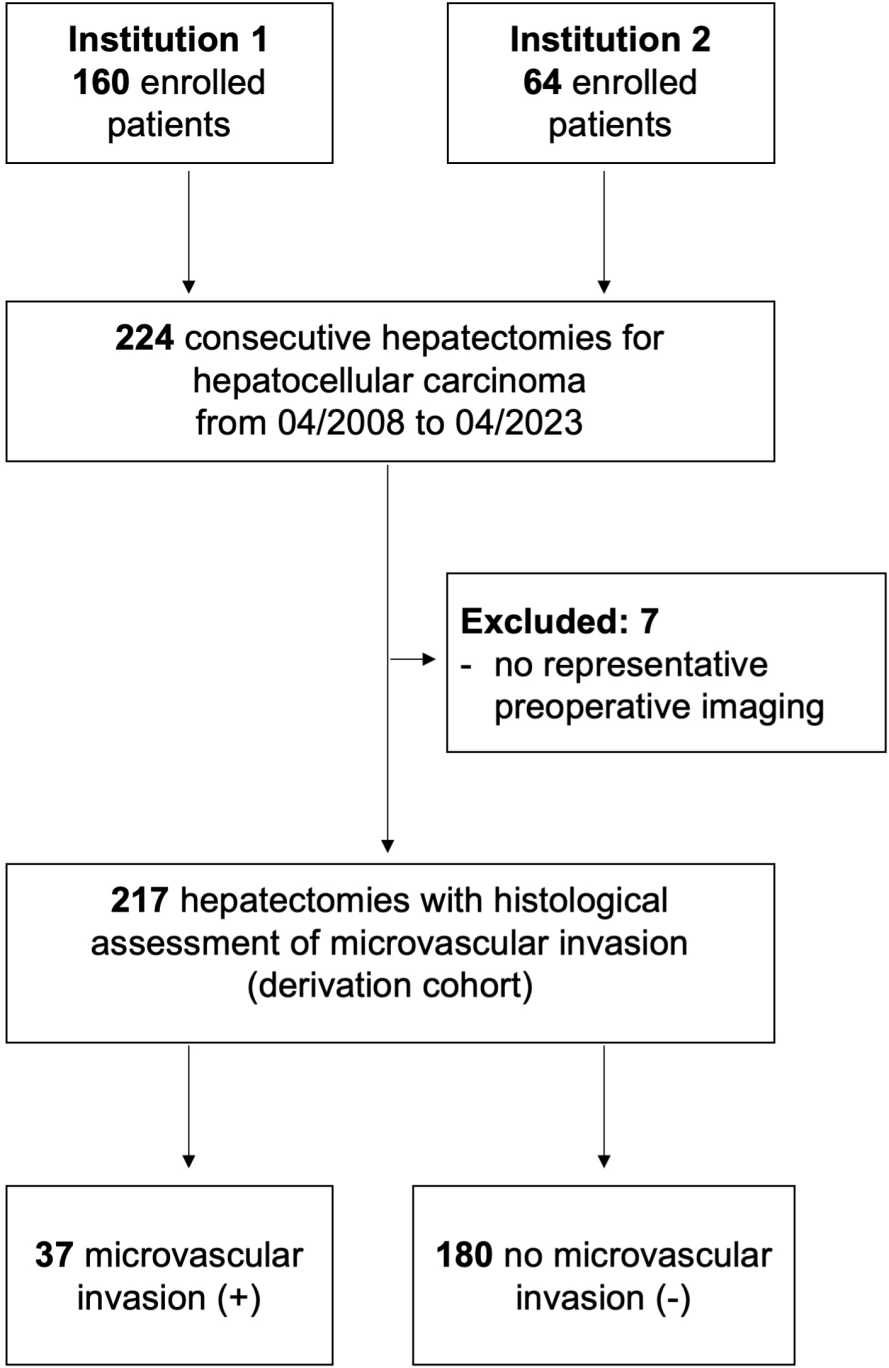
Patient flow chart.

A total of 217 patients underwent hepatectomy for hepatocellular carcinoma at both institutions during the study period (**Figure 1**). Of these, 37 patients (17%) had a histopathological positive MVI. The baseline characteristics of patients with and without MVI are detailed in **Table 1**. In the MVI-positive group, more patients showed lesions exceeding 5 cm (68% vs. 43%, P = 0.030) and required major hepatectomies (46% vs. 17%, P = 0.030) as compared to patients in the MVI-negative group. Other characteristics were well-balanced between the groups.

**Table 1:** Baseline characteristics of microvascular invasion positive and negative patients.

	MVI positive N = 37	MVI negative N = 180	P
**Age (years) ^†^**	69 (64 – 76)	70 (63 – 78)	0.829
**Sex ratio (Male: Female)**	26:11	150:30	0.104
**Etiology of liver disease**			0.744
Alcoholic liver disease	12 (32)	60 (33)	
Metabolic liver disease	20 (54)	103 (58)	
Viral hepatitis	5 (14)	17 (9)	
**Liver cirrhosis**	25 (68)	98 (54)	0.151
**Child-Pugh classification**			0.491
Child A	23 (62)	89 (49)	
Child B	2 (5)	9 (5)	
Preoperative laboratory values			
ALT, U/l	56 (65)	45 (39)	0.333
AST, U/l	74 (125)	43 (28)	0.079
Albumin, g/l	37 (6)	35 (5)	0.511
Bilirubin, mg/dl	0.8 (0.5)	0.7 (0.4)	0.319
Platelet count, x 10^9^/l	211 (99)	220 (105)	0.703
INR	1.1 (0.1)	1.1 (0.1)	0.114
**No. of lesions**			0.394
single	29 (79)	155 (86)	
oligonodular	6 (16)	15 (8)	
multinodular	2 (5)	10 (6)	
**Lesion size, mm**			0.030
30	5 (14)	40 (22)	
30 - 50	7 (19)	63 (35)	
50	25 (68)	77 (43)	
**Type of hepatectomy**			0.123
Non-anatomic resection	2 (5)	30 (17)	
Anatomic resection	35 (95)	150 (83)	
**Extent of resection**			0.007
Minor hepatectomy	17 (46)	127 (71)	
Major hepatectomy	20 (54)	53 (29)	

MVI microvascular invasion, ALT alanine aminotransferase, AST aspartate aminotransferase, INR international normalized ratio, mm millimeter.

**^†^** Values are median (interquartile range).

### Analysis of imaging risk factors for MVI

**Figure 2:**
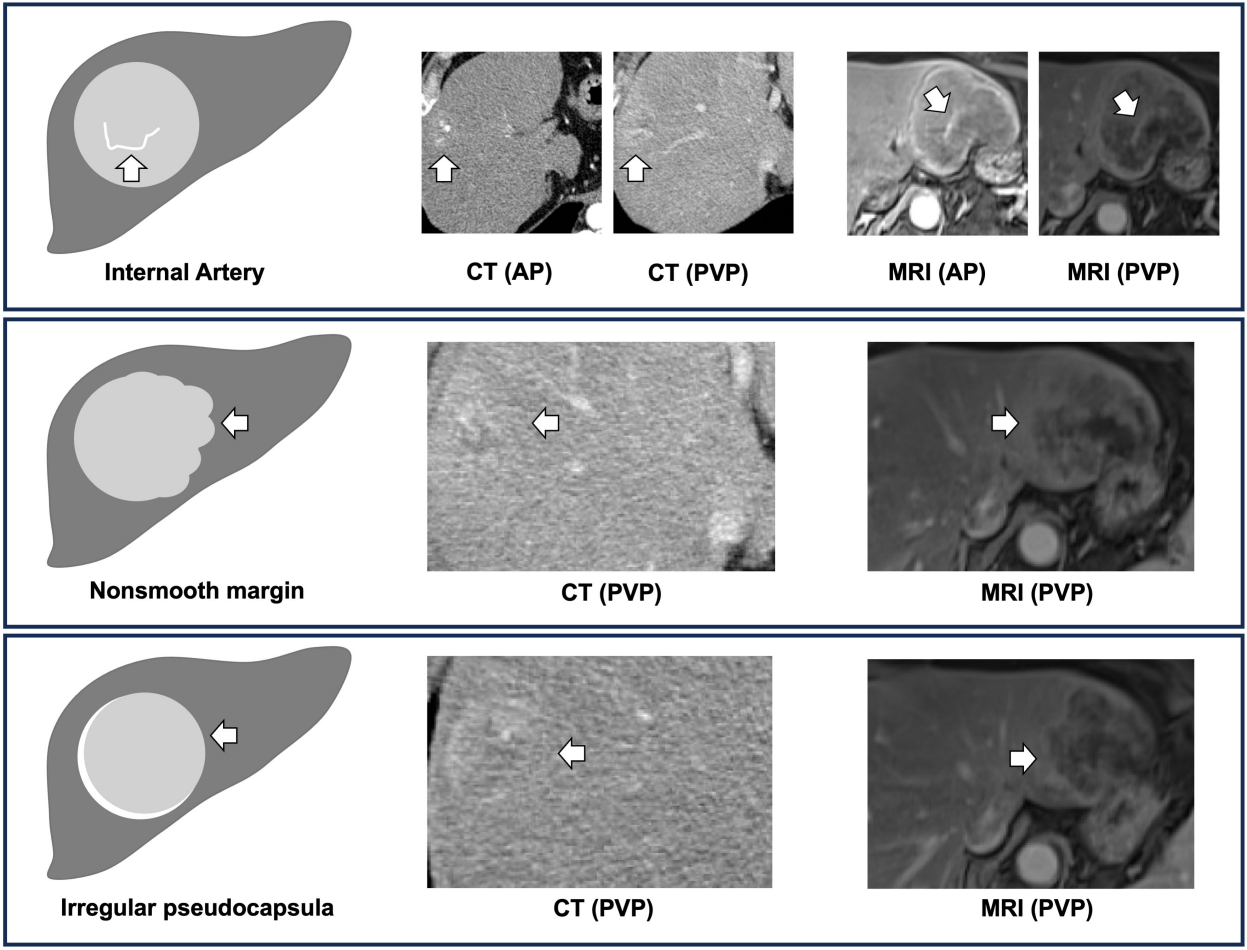
Digital biopsy features of microvascular invasion. The digital biopsy features (i.e. internal artery, nonsmooth margin, irregular pseudocapsula) are illustrated with white arrows (CT, computed tomography; MRI, magnetic resonance imaging; AP, arterial phase; PVP, portal venous phase).

To develop a digital biopsy prediction model, we initially performed a logistic regression analysis (with adjustments for age, sex, etiology of disease, size, and number of lesions) on 10 potential predictive variables, which were previously shown to be associated with MVI (**Table 2**) (8, 10, 16, 20, 21). We identified three distinct imaging features, on univariate analyses to be associated with MVI i.e., 1) internal artery (OR 29.90, P < 0.001), 2) irregular pseudocapsula (OR 4.42, P < 0.001), and 3) non-smooth peritumoral margin (OR 12.40, P < 0.001). Multivariate analysis confirmed all three features as strong independent predictors of MVI. A non-smooth peritumoral margin predisposed a 16-fold increase, while an internal artery or an irregular pseudocapsula was associated with a 10-fold and 5-fold increase of the likelihood for the histopathological diagnosis of MVI, respectively. **Figure 2** illustrates these distinct imaging features to predict MVI.

**Table 2:** Univariate und multivariate analysis of radiological factors associated with microvascular invasion.

	Univariate		Multivariate		
	OR (95%CI)	P	OR (95%CI)	β	P
Growth pattern					
Intrahepatic (Ref.)	1				
Extrahepatic	0.40 (0.10 – 1.59)	0.194	–		
Intratumoral necrosis					
Absent (Ref.)	1				
Present	2.39 (0.88 – 7.00)	0.083	–		
Intratumoral hemorrhage					
Absent (Ref.)	1				
Present	1.31 (0.59 – 2.91)	0.501	–		
Intratumoral vascularity					
Mild (Ref.)	1				
Hypervascularity	2.91 (0.99 – 8.54)	0.051	–		
Internal artery					
Absent (Ref.)	1				
Present	29.90 (10.20 – 87.90)	<0.001	10.50 (3.37 – 32.50)	2.35	<0.001
Pseudocapsula					
Well-defined (Ref.)	1				
Irregular	4.42 (1.91 – 10.20)	<0.001	4.93 (1.59 – 15.30)	1.60	<0.001
Margin smoothness					
Smooth (Ref.)	1				
Non-smooth	12.40 (4.75 – 32.10)	<0.001	16.40 (4.39 – 61.50)	2.80	<0.001
Peritumoral halo					
Absent (Ref.)	1				
Present	1.81 (0.84 – 3.90)	0.133	–		
Rim enhancement					
Absent (Ref.)	1				
Present	1.46 (0.69 – 3.08)	0.320	–		
Wedge-shape lesion					
Absent (Ref.)	1				
Present	1.70 (0.79 – 3.67)	0.175	–		

No. number, Ref. reference.

Adjustments were made for age, sex, etiology of liver disease, size and number of lesions.

### Digital biopsy model

**Figure 3:**
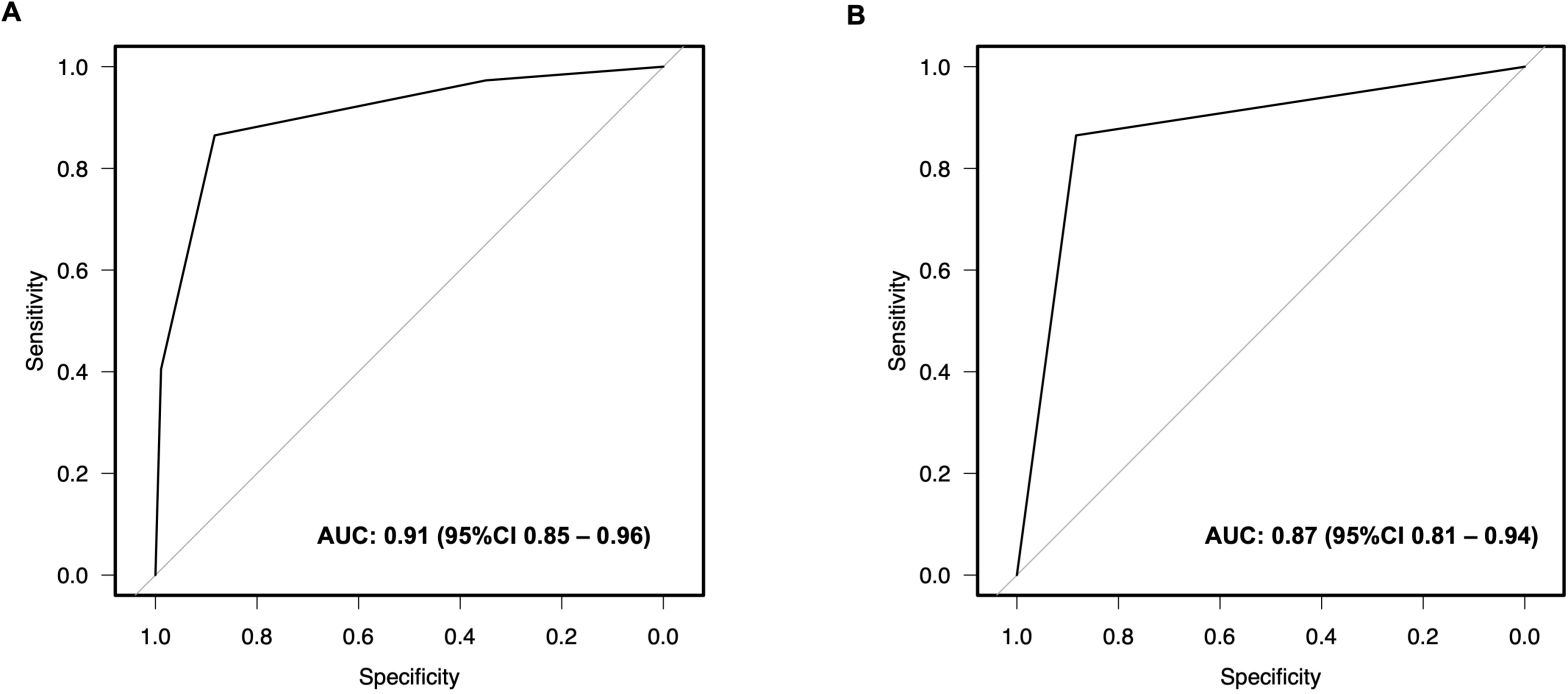
Receiver Operating Characteristics (ROC) curves of the digital biopsy microvascular invasion predictive system. The digital biopsy model with 0- to 3 points (yielded an area under the curve (AUC) of 0.91 (95% CI 0.93 - 0.99) **(A)**. An AUC of 0.87 (95% CI 0.81 - 0.94) was calculated for the digital biopsy risk model with high vs. low risk of microvascular invasion **(B)**.

In the next step, we assigned scores proportional to the β regression coefficient with a single point for each risk factor. The digital biopsy model resulted in a discrimination ability of an AUC of 0.91 (95%CI 0.85 – 0.96) to predict MVI (**Figure 3A**). Of 37 patients with histopathologically confirmed MVI, 36 (98%) had at least one point on the model. The overall MVI positivity rate was 2%, 4%, 47%, and 88% in patients with 0, 1, 2, and 3 points, respectively. Internal validation of the digital biopsy model using 1,000 bootstrap samples confirmed a high discrimination ability with a corrected AUC of 0.90. **Supplementary Figure S1A** displays the calibration plot with an excellent calibration between predicted and observed MVI frequencies. Further model metrics revealed high prediction value (Brier-Score of 0.08) and relationship between the predictors and MVI (Nagelkerke R^2^ of 0.56).

### Stratification between high and low risk MVI

To enhance the clinical utility of the digital biopsy model, we applied a threshold analysis to discriminate between high risk and low risk MVI patients. A threshold of 2 points was determined to predict a high risk for MVI with a sensitivity of 87% and specificity of 88%. Of 53 patients with 2 points on the digital biopsy risk model, 32 (60%) had confirmed MVI on histopathological analysis, compared to 5 (10%) out of 164 patients with < 2 points (**Table 3**). The discrimination analysis of this digital biopsy risk model yielded an AUC of 0.87 (95%CI 0.85 – 0.96) (**Figure 3B**). Bootstrap validation of the risk model resulted in a corrected AUC of 0.87, a Brier-Score of 0.08, and a Nagelkerke R^2^ of 0.52. The calibration plot of the risk model is shown in **Supplementary Figure S1B**.

**Table 3:** Risk classification system of digital biopsy proved microvascular invasion.

Risk classification	Total Points	Patients N = 217	MVI + N = 37	MVI rate % (95% CI)
**Low**	0	64 (29)	1 (3)	2 (1 – 8)
	1	100 (46)	4 (11)	4 (1 – 10)
**High**	2	36 (17)	17 (46)	47 (30 – 65)
	3	17 (8)	15 (41)	88 (64 – 99)

MVI + microvascular invasion positivity.

### Survival outcome

**Figure 4:**
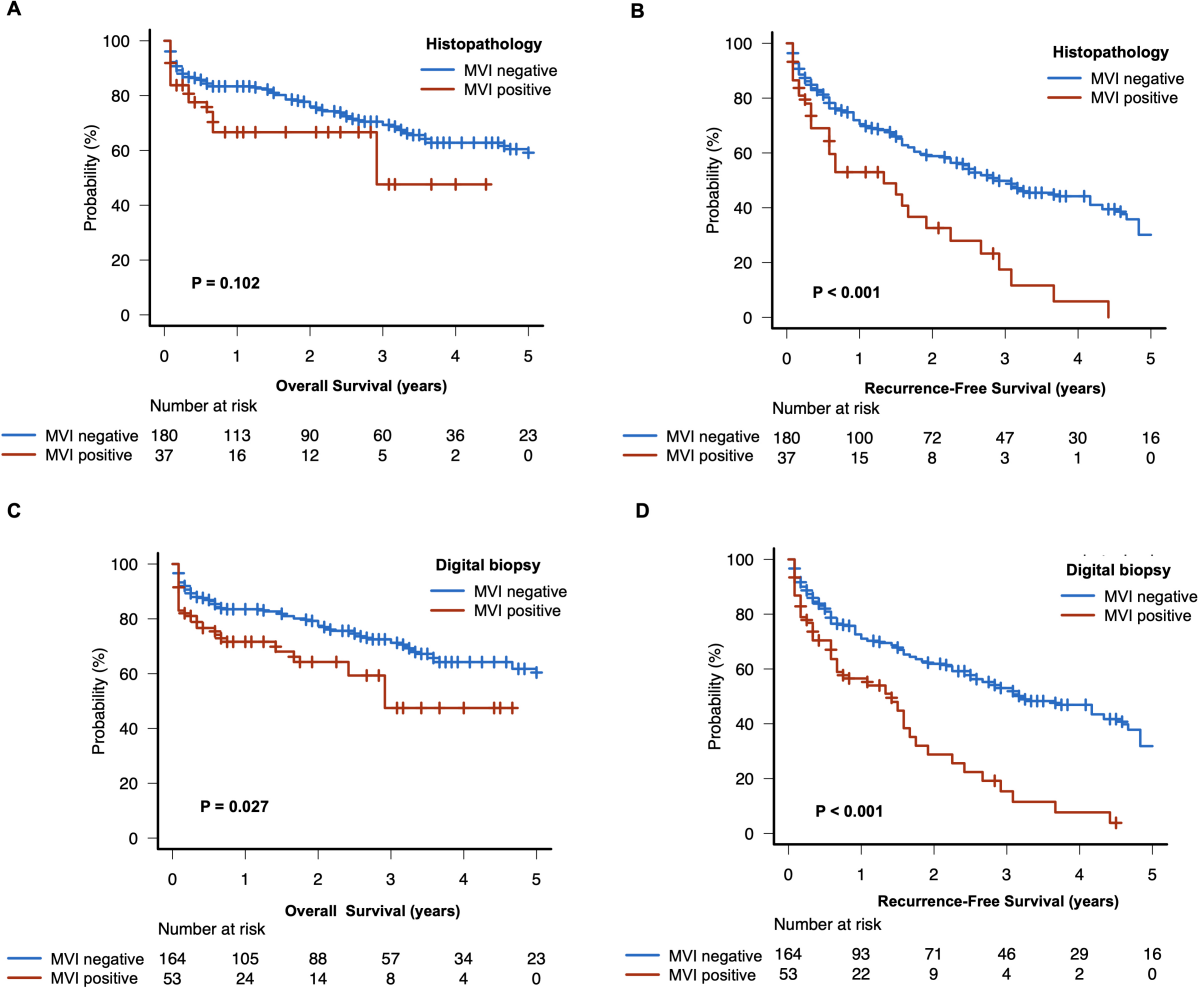
Survival plot stratified by pathologic and digital biopsy of microvascular invasion. The overall and recurrence-free survival plots of histopathological MVI **(A, B)** and digital biopsy proven MVI **(C, D)** are displayed.

The median follow-up was 20 months (interquartile range: 4 - 40). Patients with histopathological confirmed MVI had a shorter recurrence-free survival (16 months, 95%CI: 7 - 23, vs. 34 months 95%CI: 26 - 52; P < 0.001), while there were no significant differences in overall survival compared to patients without MVI (35 months, 95%CI: 8 - NA, vs. 72 months 95%CI: 60 - NA, P < 0.102) (**Figures 4A, B**). Notably, patients with high risk of MVI according to our digital biopsy risk model had both a significantly shorter overall survival and recurrence-free survival as opposed to patients with low risk of MVI (overall survival: 35 months, 95%CI: 20 - NA, vs. 75 months 95%CI: 60 - NA, P < 0.027; recurrence-free survival: 17 months, 95%CI: 7 - 21, vs. 38 months 95%CI: 30 - 55; P < 0.001) (**Figures 4C, D**).

## Discussion

In the present dual-center study, we developed a straightforward scoring system to predict MVI in patients with HCC using distinct preoperative radiologic features. Our digital biopsy model included the presence of a non-smooth peritumoral margin, intratumoral arterial enhancement, and irregular peritumoral hyperenhancement. The presence of at least two out of three radiologic features was associated with high sensitivity (87%) and specificity (88%) for predicting MVI before hepatectomy. Moreover, the survival outcomes stratified by our digital biopsy risk model achieved a significant difference of overall and recurrence-free survival in patients with predicted high risk versus low risk of MVI.

MVI is defined as the microscopic presence of cancer cells in hepatic vessels, indicating aggressive tumor biology (7). On preoperative imaging, a non-smooth peritumoral margin implies an upfront sign of tumor aggressiveness characterized by tumor protrusion into peritumoral areas (26). A meta-analysis on the predictive value of a nonsmooth peritumoral margin revealed that a non-smooth peritumoral margin is associated with a diagnostic odds ratio of >20 for MVI (27). Three other meta-analyses on the impact of different preoperative imaging features to predict MVI determined that a nonsmooth peritumoral margin is an important independent predictor of MVI (28–30). In the present study, we confirmed that this marker was the strongest predictor of MVI.

The presence of internal arteries in the portal phase is another substantial radiologic marker of MVI. Previous studies reported that internal arteries in HCC are correlated with angiogenesis and cellular proliferation which in turn results in tumor progression (31, 32). In 2007, a radiogenomic biomarker to predict MVI was developed in 28 patients with HCC and further validated in a cohort of 157 patients (29% with MVI) (20, 33). This radiogenomic biomarker was based on the correlation of two combined radiologic features (i.e., presence of internal arteries and peritumoral hypodense halo) with angiogenesis gene expression patterns and resulted in a sensitivity and specificity of 76% and 94% to predict MVI (20, 33). Some studies confirmed the predictive value of these two combined radiogenomic features (8, 34), while other studies including larger patient cohorts reported conflicting results (35, 36) and discovered an even higher predictive and prognostic value for internal arteries compared to peritumoral hypodense halo (12). Thus, in our study, we evaluated no combined imaging features and depicted a high predictive value of internal arteries for MVI, while peritumoral hypodense halo failed statistical significance to predict MVI.

Another characteristic imaging feature of advanced HCC is the presence of a radiologic tumor capsule (referred to pseudocapsula or peritumoral hyperenchancement), found in 70% of HCC cases (37). While the absence of a pseudocapsula might indicate an early HCC, an irregular or incomplete pseudocapsula is associated with MVI (diagnostic odds ratio of 1.85) according to a meta-analysis (38). In the present study, an irregular pseudocapsula was one of the three independent imaging features associated with MVI.

So far, a plethora of risk models exists in the literature, incorporating imaging features to predict MVI (11, 39, 40). However, the majority of available risk models were developed in patients with viral HCC having a high incidence of MVI. These models included multiple candidate variables in multivariate analyses based on the “rule of thumb”, without considering sample size considerations (8, 9, 12, 16). Renzulli et al. identified three “worrisome” features (i.e., nonsmooth tumor margin, the radiogenomic features (combination of internal arteries and hypodense peritumoral halo), and irregular pseudocapsula) to be associated with MVI in a total of 140 patients (64% with MVI and 6 candidate variables) with a c-index of 0.85 and 0.90 (8). However, these features were not tested in a multivariate analysis (8). Similarly, Min et al. described a diagnostic model in a total of 100 patients (39% with MVI) including four radiologic features (i.e., non-smooth margin, irregular pseudocapsula, peritumoral hyperenhancement, peritumoral hypointensity) with a c-index of 0.80, again without multivariate testing (9). Lee et al. developed a 6-point risk model including two radiologic features (i.e., peritumoral arterial enhancement, peritumoral hypointensity) and two serological biomarkers in a total of 276 patients (28% with MVI and 15 candidate variables) with a c-index of 0.87 (16). Recently, Jiang et al. presented a complex 10-point risk model that outperformed the models of Renzulli et al., Min et al., and Lee et al. in a comparative analysis. However, the risk model by Jiang et al. was developed in 319 patients (47% with MVI), evaluating 22 candidate variables in a multivariate analysis. Hence, the current risk models in literature are at high risk of statistical overfitting owing to the high number of candidate variables and the selection of MVI patients (MVI incidence ranging between 27% - 64%) (24). The major strength of our digital biopsy risk model is that we performed a formal sample size calculation and adjusted our analysis by controlling for several confounders (i.e., etiology of disease, lesion pattern, age, sex) which were not addressed in previous studies (8, 9, 16, 20, 33). Our digital biopsy risk model achieved a high discrimination value (c-index of 0.87) and yielded excellent calibration metrics. Notably, we included only three imaging features on CT or MRI, making it highly applicable in the daily routine compared to other models comprising more features with or without additional serum analyses as well as restrictions on the imaging modality (i.e., MRI or CT) (8–12). Additionally, the prognostic utility of our risk model was further proven by stratified survival analyses. Remarkably, patients with a high risk of MVI on the digital biopsy risk model reflected an even worse prognosis in terms of overall survival compared to patients with histopathologically proven MVI. This finding is in line with other reports in literature, indicating a higher prognostic performance of pretreatment radiologic features as compared to histopathological MVI (41, 42). Therefore, our risk model provides a “digital biopsy” and may represent an additional noninvasive armamentarium to facilitate personalized HCC treatment strategies and improve patient outcome. To this end, patients with high risk of MVI might benefit from neoadjuvant local (i.e., chemoembolization, radioembolization) or systemic treatment prior surgical resection. Given that patients with high risk of MVI recur more frequently, the digital biopsy model could also be helpful to stratify candidates for liver transplantation and, thus, impacting organ allocation policies.

There are some limitations to our study. This is a retrospective prognostic study with a potential selection bias. To increase the generalizability of our results, we performed a rigorous methodology with adjusted analyses, and imaging features were evaluated by local radiologists at each center. Still, our study lacks an external validation cohort, and the findings of our study need to be tested in a separate HCC population.

In conclusion, we developed and internally validated a robust and reliable prediction model of MVI.

## Data Availability

The raw data supporting the conclusions of this article will be made available by the authors, without undue reservation.
