# Supplementary figures and images for: Development and validation of a digital biopsy model to predict microvascular invasion in hepatocellular carcinoma
Source: Front Oncol. 2024 Sep 17;14:1360936. doi: 10.3389/fonc.2024.1360936 (PMC11457731; doi:10.3389/fonc.2024.1360936)

## *Supplementary Material*

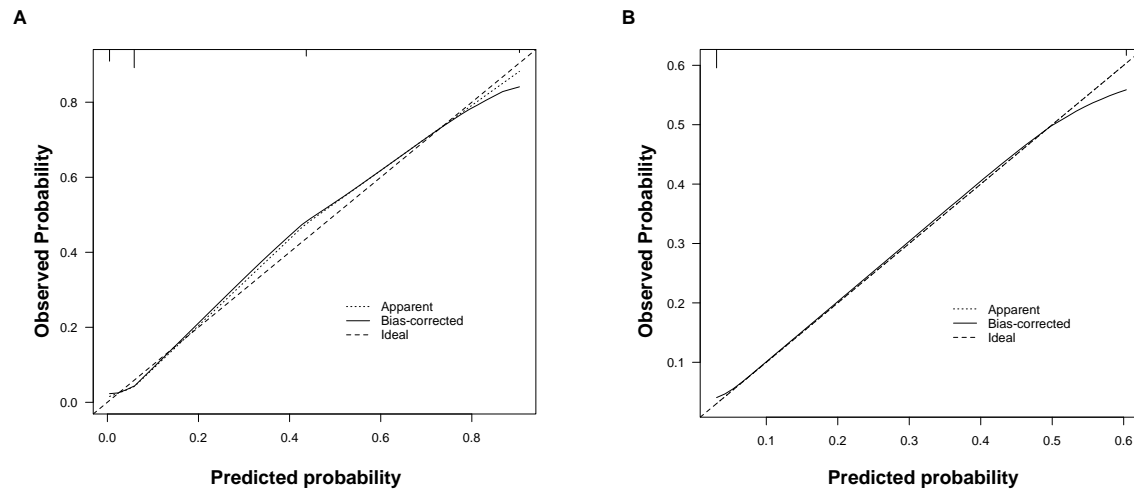

Supplement: Supplementary file 1 [file Image1.pdf]
